# Lamotrigine as an alternative treatment for paroxysmal kinesigenic dyskinesia

**DOI:** 10.1002/cns3.20017

**Published:** 2023-05-17

**Authors:** Heather Leduc‐Pessah, Asif Doja

**Affiliations:** ^1^ Department of Pediatrics, Division of Neurology, Children's Hospital of Eastern Ontario University of Ottawa Ottawa Ontario Canada; ^2^ Children's Hospital of Eastern Ontario Research Institute University of Ottawa Ottawa Ontario Canada

**Keywords:** epilepsy in women, lamotrigine, paroxysmal dyskinesia, paroxysmal dystonia, paroxysmal kinesigenic dyskinesia

Brief episodes of involuntary movement triggered by purposeful actions are characteristic of paroxysmal kinesigenic dyskinesia (PKD) and can interfere with daily life.^1^ Carbamazepine and oxcarbazepine are effective at reducing episodes but have teratogenic risks that limit their therapeutic potential.^2^ There is limited information describing alternate sodium channel blockers as first‐line therapies for PKD, and specifically there is no recommendation for the use of alternative agents for females of childbearing potential. We conducted an institutional retrospective chart review of patients with PKD seen between 2013 and 2022. Our research ethics board approved the identification of participants by diagnostic code in the electronic medical records and waived the requirement for written informed consent.

Eleven patients were identified with a confirmed diagnosis of PKD. Features of the cohort are outlined in Table 1. Ten patients were started on a sodium channel blocking agent. Five patients received carbamazepine (Table 2), with complete resolution of the movements in four patients and more than 90% reduction in the other individual. One person took phenytoin for several years before transitioning to carbamazepine. Three patients took oxcarbazepine, with a >90% reduction in events in two (Table 2). Two girls were treated with lamotrigine as a first‐line agent.

Patient 1: Onset estimated age 8 years, her episodes involve an aura (“tickling sensation”) in the foot followed by unilateral posturing of the arm or leg (left or right) +/− facial dystonia. Provoked by running or walking. Duration: 30 s. Frequency: “many”/day. Treated at age 15 with lamotrigine 25 mg daily, increased to twice daily, tolerated for 4.5 years at time last seen. Has an affected sibling and pathogenic mutation in *PRRT2*.
Patient 2: Onset age 10 years, she describes a feeling of weakness in the right side of the body, with head turn to the right, right arm posturing towards chest, and muscles feel tense or “paralyzed.” Episodes are stereotyped and consistently provoked by walking up stairs. Duration: 5 s. Frequency: “multiple”/day. Treated at age 16 with lamotrigine 50 mg daily, tolerated for 1.5 years at time last seen. Did not pursue genetic testing.

Both patients reported complete resolution of their events on lamotrigine with recurrence only with missed doses.

**Table 1:** Cohort features.

	Total *n* = 11 (%)
*Sex*	
Male	5 (45)
Female	6 (55)
*Age of onset*	
0–5	3 (27)
6–9	3 (27)
10–14	5 (45)
*Symptoms*	
Dystonia	11 (100)
Chorea	1 (9)
Other^a^	1 (9)
*Laterality*	
Bilateral	5 (45)
Unilateral (side locked)	3 (27)
Unilateral (alternating)	3 (27)
*Body part affected*	
Upper extremities	10 (91)
Lower extremities	9 (82)
Face	6 (54)
Daily occurrence	11 (100)
*Duration*	
<10 s	5 (45)
10–30 s	5 (45)
30–60 s	1 (9)
Aura	7 (64)
Genetic testing completed	9 (82)
*PRRT2* mutation identified	4/9 (44)

^a^ Patient reports associated loss of tone (limp) in the affected limb(s).

**Table 2:** Response to pharmacologic agents.

Medication	*n*	First line	Average daily dose (mg)	Reduction in episodes			Adverse effects
				100%	90%–99%	<25%	
Carbamazepine	5	4	300	4 (80)	1 (20)		2 mild^a^
Oxcarbazepine	3	3	535	1 (33)	1 (33)	1 (33)	1 severe^b^, 1 mild^a^
Lamotrigine	2	2	50	2 (100)			None
Phenytoin	1	1	50		1 (100)		None

^a^ Drowsiness and fatigue.

^b^ Low mood, depression, suicidal ideation.

We propose lamotrigine as a preferred agent in females of childbearing potential. Pharmacological management of PKD is indicated for frequent, intolerable episodes that interfere with daily life. There are no clinical trials for PKD, so physicians must rely on Class IV evidence to guide management. The literature supports the use of carbamazepine and oxcarbazepine as equivalent first‐line agents in the management of PKD.^1^, ^3^, ^4^, ^5^

Lamotrigine has been suggested as a second‐line agent for PKD with few reports of use as a first‐line agent.^6^, ^7^, ^8^ The largest cohort reported 100% attack‐free rate after four weeks of lamotrigine in 18 pre‐pubescent children.^6^ Of particular importance is the evidence that carbamazepine and oxcarbazepine can cause rare but significant fetal malformations when used in females of childbearing age, whereas lamotrigine has the lowest risk of fetal malformation.^2^ A recent meta‐analysis found a statistically significant increase in major congenital malformations with carbamazepine monotherapy (odds ratio [OR] 1.37) and oxcarbazepine (OR 1.32 *not statistically significant) compared to lamotrigine (OR 0.96).^2^

The need for an alternative agent in this population is not adequately addressed in the literature. Our experience suggests that lamotrigine is an effective agent in adolescent post‐pubertal patients and may be a safe and effective option for females of childbearing potential. Further studies with larger cohorts will be required to investigate lamotrigine's efficacy and tolerability as a first‐line agent for PKD and to better understand the effect of pregnancy on PKD and on lamotrigine therapy.

## Author Contributions

**Heather Leduc‐Pessah**: Conceptualization (supporting); methodology (lead); data curation (lead); writing—original draft (lead); writing—review and editing (equal). **Asif Doja**: Conceptualization (lead); methodology (supporting); writing—review and editing (equal); supervision (lead).

## Conflict of Interest Statement

The authors declare no conflicts of interest.
